# Hepatitis C Viremia Patterns in Incident Hepatitis C Infection and One Year Later in 150 Prospectively Tested Persons Who Inject Drugs

**DOI:** 10.1371/journal.pone.0097022

**Published:** 2014-05-15

**Authors:** Marianne Alanko Blomé, Per Björkman, Vilma Molnegren, Peter Höglund, Anders Widell

**Affiliations:** 1 Infectious Disease Unit, Division of Clinical Sciences, Lund University, Malmö, Sweden; 2 Department of Clinical Microbiology, Division of Laboratory Sciences, Lund University, Malmö, Sweden; 3 Medical Statistics and Epidemiology Unit, Research and Development Centre Skåne, Lund, Sweden; Inserm, U1052, UMR 5286, France

## Abstract

**Objectives:**

To assess HCV viremia levels just before, during and one year after anti-HCV seroconversion in people who inject drugs (PWID).

**Methods:**

PWID enrolling into a needle exchange program in Malmö, Sweden, 1997–2005 constituted the source population. Sera were obtained at enrolment and at approximately 3–4 monthly intervals afterwards, and were initially tested for anti-HIV, HBsAg/anti-HBc and anti-HCV and thereafter for markers previously negative. Seroconversion to anti-HCV had occurred during the study period in 186 out of 332 seronegative subjects. In these anti-HCV seroconverters, quantitative HCV RNA PCR was retrospectively performed on frozen sera to determine viremia levels in the last anti-HCV negative, the first anti-HCV positive and in one year follow-up samples.

**Results:**

Among 150 subjects seroconverting to anti-HCV with samples available from all three defined time-points, eight different patterns of viremia were observed. Spontaneous clearance at one year was noted in 48 cases (32%) and was associated with female gender (p = 0.03, CI 0.17–1.00). In 13 cases HCV-RNA was not detected in any study sample. Among 61 subjects with pre-seroconversion viremia, viral load was significantly higher in the pre-seroconversion samples compared to subsequent samples. For the whole group, viral load declined to undetectable levels at seroconversion in 28% of cases (but with recurrent viremia in 15%).

**Conclusions:**

Different patterns of HCV RNA kinetics were observed among PWID with documented seroconversion to anti-HCV. The frequently observed absence of detectable HCV RNA in the first anti-HCV positive sample (irrespective of subsequent viremia) demonstrates the importance of repeated sampling and RNA testing for determination of the outcome of acute infection.

## Introduction

Infection with hepatitis C virus (HCV) is a major health problem; the global prevalence is estimated to have increased from 122 million to 185 million between 1990 and 2005, with regional variations ranging from <1.5% to >3.5% [1]. Egypt has an exceptionally high HCV prevalence (14.7% of the population), compared to about 0.5% of the Swedish population (9 millions) [2], [3]. In most cases (60–85%) HCV infection becomes chronic and a high proportion develops progressive fibrosis [4]. Subjects with HCV viremia constitute the reservoir for ongoing transmission, especially among the group most at risk for HCV infection in many countries – people who inject drugs (PWID) [5]. Due to the mild clinical presentation, acute HCV infections are often not diagnosed when they occur. Instead HCV infections are usually detected in persons with unexplained liver enzyme elevations or in subjects participating in targeted screening programs; obviously leading to heterogeneity in case definitions of acute or recent HCV infection [6]. Thus, in many cases, the first test leading to a diagnosis of HCV infection is often obtained several years after seroconversion [7]. For PWID, hepatitis C is frequently first detected upon inclusion in needle exchange programs (NEP), in opiate substitution therapy or in prison settings [8]–[10].

For these reasons, the kinetics of viremia during acute infection is difficult to investigate in a representative sample of PWID, who rarely seek medical care in association with HCV seroconversion. One prison-based study among incarcerated inmates showed that pronounced fluctuations of HCV RNA levels (>1 log_10_) were common among persons undergoing seroconversion to anti-HCV, as opposed to the pattern found in chronically infected patients [11]. In general, up to 80% of acute HCV infections are asymptomatic and rarely detected; hence, most data on viremia levels in incident HCV infections are based on symptomatic cases [12]–[14]. The course of symptomatic acute hepatitis C may differ from that in asymptomatic infection, due to the presence of a more pronounced inflammatory reaction which increases the chance of viral clearance [15], [16]. The process of HCV clearance is complex, and has been associated with both viral and host genetic factors [17]. A higher chance of spontaneous clearance has been associated with female gender, IL28B polymorphism, lower age, high viral load on first testing and the infectious dose [18]–[20]. Not surprisingly, spontaneous HCV clearance is less common in subjects with immunosuppressive conditions such as after organ transplantation, with HIV infection, or both [21]–[23].

In this study, we have assessed the course of HCV viremia in incident HCV infection in a cohort of PWID attending a NEP with regular anti-HCV testing and a low HIV prevalence. HCV RNA levels in PWID with documented anti-HCV seroconversion were tested at three time points: before seroconversion, at seroconversion, and one year following seroconversion.

## Materials and Methods

### Setting

The NEP at the Department of Infectious Diseases at the University Hospital of Skåne in Malmö, Sweden, was started in 1987. This NEP is estimated to cover around 70% of all PWID in the uptake area [24]. Upon entry to the NEP the participants are requested to be tested for HIV and subsequently at three-monthly intervals. In addition, regular testing for hepatitis B (HBV) and HCV is strongly recommended, and is also accepted by a majority of participants. The first serum sample obtained is analyzed for anti-HIV, anti-HAV, HBsAg, anti-HBc, and anti-HCV. All participants negative for HBV markers are offered vaccination against HBV. Later follow-up samples are only tested for the markers that were previously negative. HCV infection is serologically confirmed by recombinant immunoblot testing. After testing the remaining portion of each serum sample is stored at −20°C in a biobank.

In a recent study, we reported the incidence of HIV, HBV and HCV among new participants, registered in the NEP from 1997 throughout 2005 [25]. In summary, HIV prevalence and incidence was low (baseline prevalence 0.12% and incidence 0.08/100 person years at risk [pyr] among 831 subjects), whereas previous exposure to HBV was found among 28% (linked with a HBV incidence of 3.4/100 pyr). Previous exposure to HCV infection was seen in 60.0% and a high proportion, 186 of 332 anti-HCV negative persons at baseline (first blood sample at enrolment in the NEP) were found to seroconvert to anti-HCV during follow-up, resulting in an incidence of 38.3/100 pyr during the time period 1997–2005. To further clarify the time point of incident HCV infection, we used frozen sera in our serum biobank to retrospectively determine presence of HCV RNA (by TaqMan PCR) on all last anti-HCV negative samples. The results (interpreted qualitatively as infected/non-infected) were included in our first report. By this combined procedure of serology and PCR the incidence of HCV infection after starting NEP could be corrected to 31/100 pyr, excluding 37 participants who were already viremic, yet anti-HCV negative, at enrolment. The incidence rate of hepatitis C did not change significantly during three-year intervals over the 9 year study period.

### Participants in current study

For the present study, only participants from the original study with documented HCV seroconversion were eligible for inclusion. The HCV RNA data generated from the last anti-HCV negative and the first anti-HCV positive samples (irrespective of detectable viremia), as well as a sample drawn about one year later, were interpreted both qualitatively and quantitatively. Complete series of these three samples were available for 150 persons from a total of 186 subjects with documented anti-HCV seroconversion. For 32 of the remaining 36 participants one year follow-up samples were not available (due to death in 4 subjects) and in 4 cases the seroconversion samples were exhausted. HCV RNA testing was done by Roche COBAS AmpliPrep/COBAS TaqMan HCV Test as described by the manufacturer. Since serum volumes were limited, all testing was done at 1/10 dilution in negative serum, hence test sensitivity was decreased tenfold. Results exceeding 150 IU/mL were considered positive. For the purpose of statistical analysis, cases with a positive but non-quantifiable PCR signal (below 150 IU/mL) were categorized as having HCV RNA levels of 149 IU/mL, and those completely non-reactive/negative one log_10_ lower (<15 IU/mL). Genotyping of the viral strains was performed by amplification and sequencing of a part of the HCV NS5B gene as described by Abdel Hamid et al [26].

### Data analysis/Statistical methods

Categorical variables were expressed as frequencies with percentages. Continuous variables were presented as median and 25% and 75% interquartile range of log_10_ -transformed values. Comparisons of viral load within each subject between time-points were analyzed with Wilcoxon signed rank test. For categorical variables the exact binomial test was used.

### Grouping of viremia patterns

With three longitudinal samples from these seroconverting subjects (defined as documented absence of anti-HCV, followed by at least two anti-HCV-positive samples) and any combination of HCV RNA, eight qualitative RNA patterns were possible; pattern A was thus negative (−) to negative (−) to negative (−) regarding viremia; pattern B − to + to −; pattern C − to − to +; D − to + to +; E + to − to −; F + to + to −; G+ to − to +; H + to + to +. Spontaneous viral clearance was defined as the absence of viremia in a follow-up sample drawn approximately 12 months after the appearance of anti-HCV.

### Sampling intervals

The median follow-up time in NEP for the anti-HCV seroconverters throughout the study period was 43 months, during which they were tested in median 7 times. Although blood sampling was recommended every 3 months, several participants did not come for phlebotomy at exact intervals, and the median time between the last anti-HCV negative and the first anti-HCV positive samples was 131.5 days (IQR 106, 216). The median time between the first anti-HCV positive and the follow-up samples was 412 days (IQR 366, 491).

To further focus on kinetics during the serological window period (with a duration of 2–3 months as indicated by studies in source plasma donors [27]), we also performed separate analyses on pre-seroconversion viremic participants with incrementally shorter time intervals between their last anti-HCV negative and first anti-HCV positive samples.

### Ethical considerations

The study was approved by the Regional Research Ethics Committee in Lund (no 195/2005). On each sampling occasion, NEP participants are asked whether they allow biobank storage of the remaining serum sample for future research testing. After approval by the Regional Research Ethics Committee, the purpose and the procedure of our study were announced on posters in the NEP and in advertisements in the local press, including a widely distributed free daily newspaper. An opt-out strategy was used, assuming consent for NEP participants who did not actively object to study inclusion. While all virological testing was done with access to a personal identity number, all subsequent handling of patient data was done under code.

## Results

### Baseline characteristics

The gender distribution among the 150 persons in our cohort of incident HCV infection was 119 (79%) males, while the median age at the first anti-HCV positive sample was 27 years (range 21–53). Thirty-four percent reported injecting heroin and 25% amphetamine, while the majority used both drugs (41%). The median duration of drug use prior to HCV infection was 2 years for heroin (range 0–14 yrs) and 3 years for amphetamine (range 1–30 yrs) users, respectively. Prior exposure to HBV (defined as presence of anti-HBc) was observed in 16 persons, whereas no study participant had ongoing HBV or HIV infection.

### Patterns of HCV viremia

**Figure 1:**
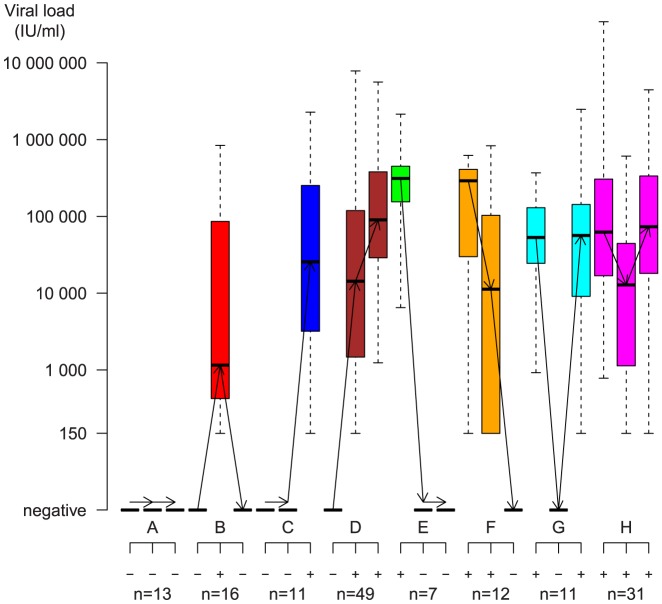
Viral kinetics in the eight different groups (A through H) observed. Groups are defined as described in Materials and Methods, depending on if viremia was detectable or not and indicated by + or −. Viral loads, presented as boxplot diagrams, in different colour for each group (A = black, B = red, C = dark blue, D = brown, E = green, F = orange, G = cyan/light blue, H = magenta) show median, 25% and 75% interquartile range and outliers. For each patient group, the first time point/column represents the last anti-HCV negative sample, the second first anti-HCV positive and the third the one year follow-up. For each group A-H the number of subjects is shown. Viral loads are presented in a log_10_ scale, and the samples that were RNA positive but below quantifiable as 149 IU/mL, to distinguish from samples where RNA was not detected, which were set one log_10_ lower at 15 IU/mL.

Characteristics of patients following each of the eight pre-defined viremia patterns are shown in figure 1. Overall, no viremia was detected in the last anti-HCV negative sample in 89/150 (60%) cases (patterns A–D), whereas viremia was detected in this sample in 61/150 (40%) cases (patterns E–H), thus in the serological window phase. Spontaneous viral clearance at one year was observed in 48/150 (32%) cases (patterns A–B, E–F). In 22/150 (15%) cases HCV RNA was not detected in the seroconversion sample, but was detectable in the follow-up sample (patterns C and G).

**Figure 2:**
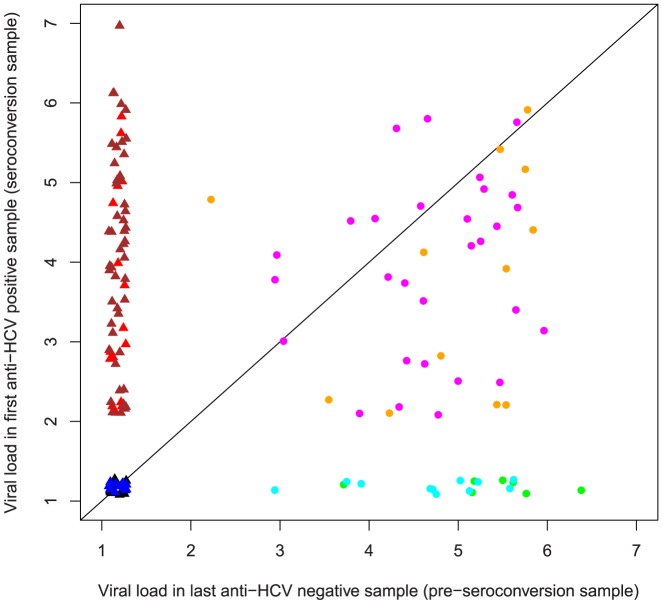
Pairwise comparison of individual viral loads in the pre-seroconversion versus seroconversion samples. Patients (n = 61) with detectable viremia already in the pre-seroconversion sample are shown by circles, and those non-viremic (n = 89) in the last anti-HCV negative by triangles, the latter group not yet showing any sign of being infected. Patients in each group A–H are displayed in the same colours as in figure 1(A = black, B = red, C = dark blue, D = brown, E = green, F = orange, G = cyan/light blue, H = magenta). Viral titres are shown in a log_10_ scale, statistical analysis by exact binomial test. Statistical analysis was also done excluding group G (n = 11, pattern + to − to +), where re-emergence vs re-infection was difficult to establish. When the total cohort (n = 150) was included, viral load was significantly lower or equal to in the pre-seroconversion sample (p<0.001, CI 0.57–0.73) due to the majority still being non-viremic/non-infected. For the subgroup with pre-seroconversion viremia, viral load was instead significantly lower in the seroconversion sample than in the pre-seroconversion sample (p<0.001, CI 0.74–0.93). Excluding group G, values remained similar for the whole cohort (now n = 139, pre-seroconversion viral load lower than seroconversion viral load, p = 0.003, CI 0.54–0.71) as well as for the subgroup viremic in the window phase (now n = 50, seroconversion viral load lower than pre-seroconversion, p<0.001, CI 0.69–0.91).

**Figure 3:**
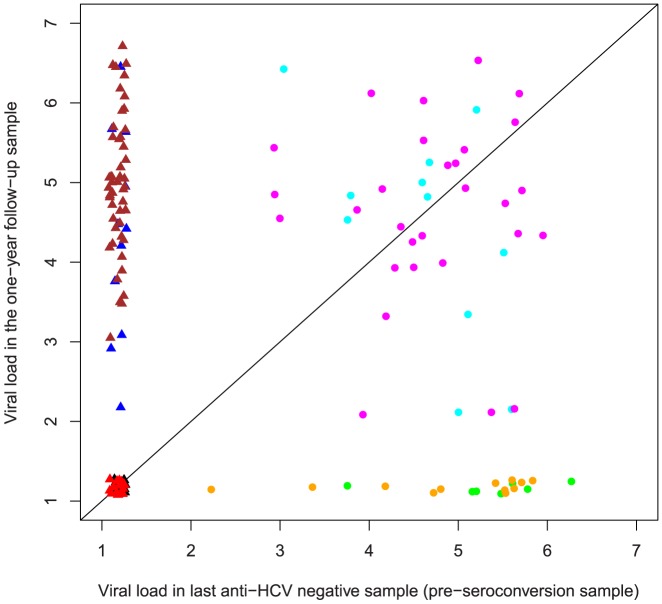
Pairwise comparison of individual viral loads in the pre-seroconversion versus one year follow-up samples. Methodology as in figure 2. Viral load was significantly lower or equal to in the follow-up sample (p<0.001, CI 0.66–0.81) for the whole group (p<0.001, CI 0.67–0.82 when excluding group G). For the subgroup of pre-seroconversion viremic values were p = 0.04, CI 0.24–0.49 (p = 0.007, CI 0.18–0.45 when excluding group G).

**Figure 4:**
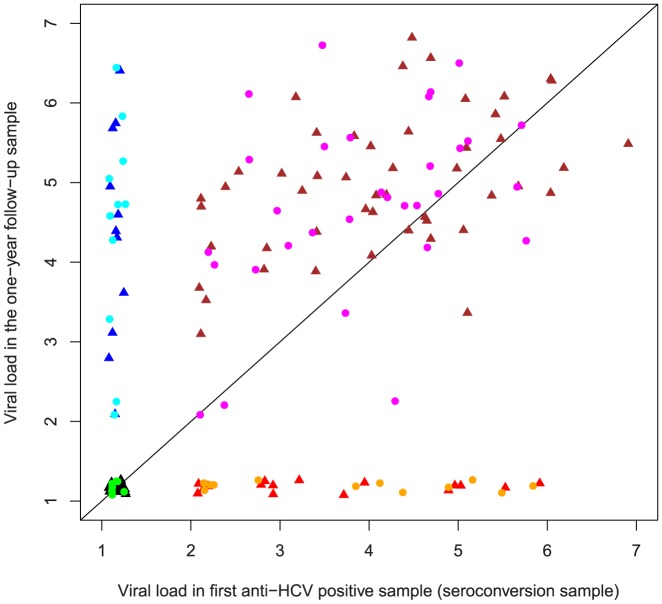
Pairwise comparison of individual viral loads in the seroconversion versus one year follow-up samples. Methodology as in figure 2 and 3. Viral load was significantly lower or equal to in the seroconversion sample compared to the one year follow-up sample for the whole cohort(p<0.001, CI 0.63–0.78), as well as in the subgroup of pre-seroconversion viremic (n = 61, p = 0.002, CI 0.57–0.81). Excluding group G the values for the whole cohort were similar (p<0.001, CI 0.60–0.76), but became less significant for the pre-seroconversion viremic subgroup p = 0.065, CI 0.49–0.77.

Since blood sampling was performed at reasonably regular time intervals and not directed by suspected exposure nor symptoms, the last anti-HCV negative bleeds were dichotomous regarding whether the participants at that time point were infected or not. This was taken in account when viral loads at different time points were compared. Of the 150 pre-seroconversion samples 61 were viremic for HCV (labeled by circles in figures 2–4) whereas the rest assumingly were obtained before the appearance of viremia (labeled by triangles in figures 2–4). Three pairwise comparisons of HCV RNA loads were made (last anti-HCV negative versus first anti-HCV positive, figure 2), (last anti-HCV negative versus one year follow up, figure 3), (first anti-HCV positive versus one year follow up, figure 4) and levels of significance were calculated both for all 150 subjects and also for the subset of 61who were viremic at onset.

Using exact binomial test with the null hypothesis that each proportion would be 0.5 (half of the patients would increase and half would decrease in viral load), viral load for the whole group (n = 150), was significantly lower in the pre-seroconversion sample than in the seroconversion sample (p<0.001, CI 0.57–0.73) since the majority were non-viremic/non-infected at this time (figure 2). However, for the subgroup with pre-seroconversion viremia, the opposite pattern was seen; viral load was instead significantly lower in the seroconversion sample than in the pre-seroconversion sample (p<0.001, CI 0.74–0.93). Since persons showing pattern G (n = 11, pattern + to − to +) could theoretically have re-infection with another HCV strain instead of re-emergent viremia with the original virus, we also made a set of separate calculations with this group excluded from the analysis; similar results were obtained as for the whole cohort (now n = 139, pre-seroconversion viral load lower than seroconversion viral load, p = 0.003, CI 0.54–0.71) as well as for the subgroup viremic in the window phase (now n = 50, seroconversion viral load lower than pre-seroconversion, p<0.001, CI 0.69–0.91).

Comparing viral load in pre-seroconversion samples versus the one year follow-up sample (figure 3), viral load was significantly lower or equal to in the follow-up sample (p<0.001, CI 0.66–0.81) for the whole group (p<0.001, CI 0.67–0.82 when excluding group G). For the subgroup of pre-seroconversion viremic values were p = 0.04, CI 0.24–0.49 (p = 0.007, CI 0.18–0.45 when excluding group G).

Also, viral load was significantly lower or equal to in the seroconversion sample compared to the one year follow-up sample (figure 4) for the whole cohort (p<0.001, CI 0.63–0.78), as well as in the subgroup of pre-seroconversion viremic (n = 61, p = 0.002, CI 0.57–0.81). Excluding group G the values for the whole cohort were similar (p<0.001, CI 0.60–0.76, but became less significant for the pre-seroconversion viremic subgroup p = 0.065, CI 0.49–0.77).

### HCV RNA kinetics in subjects with more narrow sampling intervals

**Figure 5:**
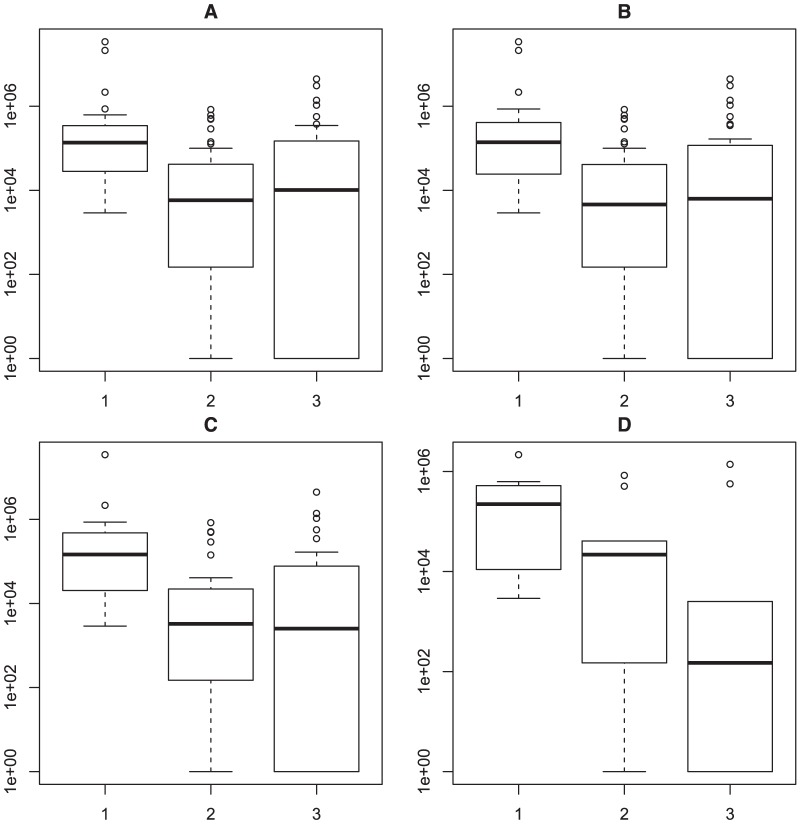
Sequential viral loads in the 50 patients identified during the serological window phase (group G, n = 11, excluded). To meet the variability in time lags between last anti- HCV negative to first anti-HCV positive, we compared the median viral load at increasingly shorter lag phases between the first two samples (T). (Panel A. T = 182 days, n = 47, p<0.001; panel B. T = 152 days, n = 43, p<0.001; panel C. T = 122 days, n = 35, p<0.001; panel D. T = 92 days, n = 11, p = 0.123). Analysis by Wilcoxon signed rank test. Figures 1, 2, and 3 on the X-axis refer to samples drawn pre-seroconversion, seroconversion and 1 year follow up, respectively.

To further focus on the course of viremia in subjects with detectable HCV RNA in the pre-seroconversion sample (group G excluded), HCV viral loads were compared in the last anti-HCV negative but viremic versus first anti-HCV positive samples using an incrementally shortened time interval between these two sampling time points (figure 5). Statistically significant declines (1 log_10_; p<0.001) were observed when the time intervals were set to 182 (n = 47), 152 (n = 43) and 122 (n = 35) days, respectively, but waned in statistically with the shorter time lags (92 days, n = 11), the latter due to low patient numbers.

### Persistent HCV viremia during the follow-up period

In their last anti-HCV negative samples, 61 persons had detectable HCV-RNA, with a median viral load of 5.0 log_10_ (range 2.17–7.54). Among these, 42 were viremic in the one-year follow-up sample. HCV RNA was detected in 102 seroconversion samples (43 from those with pre-seroconversion viremia). In the majority of persons with pre-seroconversion viremia, viral load had declined in the seroconversion sample, with a median of 1.9 log_10_ (pattern F from 4.72 to 3.52, pattern H, 4.75 to 3.78) and was undetectable in 18 subjects (pattern E, G). Only nine of 61 subjects (14.6%) had a higher viral load (increasing in median 4.47 log_10_) in the first anti-HCV positive sample than in the pre-seroconversion sample. In order to assess a potentially longer duration of pre-seroconversion viremia, we also tested the sample drawn prior to the pre-seroconversion sample, available from 39 individuals in this group (obtained at a median of 4 months before the study pre-seroconversion sample). However, HCV RNA was not detected in any of these samples.

### Viral clearance at one year post-seroconversion

Spontaneous viral clearance (defined as absence of HCV RNA in the sample obtained one year after seroconversion) was observed in 48 persons; among these, 13 were persistently non-viremic in all our study samples (pattern A). Sixteen of those who cleared their HCV infection were only viremic in the seroconversion sample (pattern B). Seven had undetectable HCV RNA in both the seroconversion and one-year follow-up samples (pattern E), whereas 12 of those who cleared the infection were viremic in both pre-seroconversion and seroconversion samples (pattern F).

Among factors potentially associated with HCV clearance, only female gender was statistically significant (p = 0.03, OR 0.412, CI 0.17–1.00) and we did not find any association with age, duration of drug use or HCV genotype. Nor did we observe a more pronounced decline of viral load between the pre-seroconversion and the seroconversion samples among women with pre-seroconversion viremia who subsequently cleared the virus compared to men with similar characteristics (data not shown).

### Genotype distribution

Genotyping of viral strains was possible in 106/150 subjects. In 19 cases the genotyping was impossible due to undetectable viremia in all samples or a persistently low viral load (15–150 mIU/mL). For 25 cases with detectable viremia we were unable to identify the genotype, neither by NS5B nor HCV core gene sequencing. Overall, the following genotypes were identified among 106 cases: 1a 49 (46.2%), 1b 4 (3.8%), 2b 11 (10.4%), 3a 42 (39.6%). No difference in genotype distribution with regard to patterns of HCV viremia was observed.

Among the 103 individuals with viremia on multiple occasions during follow-up (patterns D, F, G, H), genotyping of two separate samples from the same individual was only possible to perform in 7 cases (due to a freezer failure after the quantitative PCR and first genotyping had been conducted). In 6 of these 7 cases, the same genotype was found in the second sample, whereas only one showed a different genotype (switch from 2b to 1b).

### Incidence of co-infection or super-infection with HIV and HBV

No HIV seroconversion was observed. A small number (n = 5) of HBV infections occurred (all male, age range 22–36 years). Two were HBsAg positive in the first anti-HCV positive sample; two became HBsAg positive in samples taken three months later, suggesting infection with both viruses very closely in time. One person became HBsAg-positive in the follow-up sample 13 months later. Among these 5 persons, 4 had undetectable HCV RNA in the one-year follow-up sample (and thus fulfilled our criteria for HCV clearance). Chronic hepatitis B (defined as persistence of HBsAg for more than 6 months) developed in 3 of these subjects, whereas two became HBsAg negative. It must be noted that HBV vaccination was offered to all participants without serological signs of HBV exposure or previous immunization as soon as possible after NEP enrolment.

## Discussion

In this large cohort of PWID with incident HCV infection, different longitudinal patterns of HCV viremia were found. HCV RNA profiles were based on three sequential serum samples starting from before the time of documented seroconversion until one year after the appearance of anti-HCV. Viral load was highest in those cases that manifested positive HCV RNA in samples obtained prior to the development of antibodies, declined in median with 1.9 log_10_ in the first anti-HCV positive samples, and rose again in the follow-up samples to levels lower than prior to antibody seroconversion. Viral load was significantly higher in the pre-seroconversion sample than in the seroconversion sample (p<0.001, CI 0.57–0.73). A more pronounced decline of HCV viremia within the first months following infection has been reported among female PWID (but not male), with subsequent viral clearance [28]. In contrast, we did not find that the pattern of early HCV kinetics influenced the chance of spontaneous clearance (for the whole group or for each gender) – among 42/150 (28%) persons with undetectable HCV viremia in the seroconversion sample, 22/150 (15%) had detectable HCV RNA at the one-year follow-up.

The early decline of viral load, sometimes to undetectable HCV RNA levels around anti-HCV seroconversion can lead to misinterpretations in diagnostic algorithms if only based on a single HCV PCR conducted on the same serum sample that was first found anti-HCV positive. In most cases such an algorithm will correctly identify the HCV status of the patient in samples from low risk individuals who rarely are sampled in the acute stage – however the same algorithm may be completely misleading if applied among persons with ongoing risk behaviour such as PWID. Our findings highlight the importance of repeated sampling and testing, for viral RNA in anti-HCV-positive patients. In addition, prospective screening procedures would benefit from both antibody and viremia testing in parallel, in particular in high risk settings.

We diagnosed incident HCV infection by repeat screening and by performing retrospective PCR testing on biobank samples obtained at regular intervals, and which were selected for this study based on documented antibody seroconversion, and not guided by clinical signs of acute hepatitis. The rate of spontaneous viral clearance (32%) was, however, similar to clearance rates reported in subjects with symptomatic acute infection. Slightly higher rates of spontaneous viral clearance (42%) of newly acquired HCV infection among PWID has also been reported [29]. Comparison between the groups regarding age at seroconversion, gender and viral genotype showed no other significant differences other than a greater chance for women to spontaneously clear the virus (p = 0.03, OR 0.412, CI 0.17–1.00). The median age at seroconversion was relatively low in our cohort (27 years), which has been suggested as a favourable factor regarding spontaneous clearance [30]. The majority of the viral strains were either genotype 1a or 3a, as has been previously shown to be the most prevalent among Western PWID [31].

One inherent difficulty in studying HCV infection in PWID (in contrast to transfusion recipients or persons infected through needle-stick injuries) is that the time-point of exposure leading to infection is usually impossible to determine, and the fact that potential multiple exposure events are rather the rule than the exception. Even for subjects with access to a NEP, continuing injection drug use poses repeated risks of exposure to HCV, as both the previously observed incidence data from the Malmö NEP and other reports indicate [25], [32], [33]. Another recent longitudinal study among young PWID with ongoing risk behaviour found that several subjects with signs of spontaneous viral clearance had subsequent detectable viremia with the original viral strain (suggesting recurrence rather than reinfection) [34]. Both re- and superinfection were however quite frequent among active PWID in the Amsterdam Cohort [35]. Unfortunately we could not investigate the rate of re-infection in our material, due to loss of serum RNA for genotyping due to a freezer failure, occurring after the quantitative and initial typing studies had been completed. Thus consecutive genotype results were only obtained from 7 cases, with one observed switch from the baseline genotype. This could likely be explained by re-infection, where ongoing injection drug use is a clear risk [36]. Statistical analysis of the kinetics of viremia over time were performed both with and without group G (n = 11, pattern + to − to +, figures 2–5, data including group G not shown in figure 5), where re-emergence versus re-infection could not be differentiated, results being slightly affected by this in the subgroup with pre-seroconversion viremia. Switch of genotype has been described to occur only rarely among Norwegian PWID [37], whereas Australian findings indicate a higher rate of genotype change among PWID [38]. Possibly more sensitive methods might have revealed the presence of several genotypes at baseline. Even if the same genotype was found in both samples, this does not exclude that the viruses were heterologous or reinfection by the same genotype.

A strength for the present study was the access to a large material with longitudinal sample series (from prior to HCV seroconversion as well as follow-up samples) from PWID, in a setting with a very low HIV prevalence, which enabled us to study patterns of HCV viremia not influenced by HIV co-infection. Furthermore, cases of incident HCV infection were identified by routine regular testing among PWID enrolled for needle exchange. By sharpening the criteria of observation intervals it was possible to measure the kinetics in the pre-seroconversion to conversion period (figure 5).

However, studying NEP participants involved some limitations. Blood sampling sometimes followed the irregular schedule of people actively injecting drugs, with periods in custody, jail and also drug free periods. Shorter sampling intervals according to a more strict study protocol would certainly have described the viral kinetics around seroconversion in greater detail, but would be infeasible in the routine testing at the NEP setting both for the participants and the staff. Follow-up samples were not available for all seroconverting subjects, some of whom had died from overdose or trauma. HCV RNA testing was done retrospectively. Study samples might have been affected by storage at −20°C, instead of -70°C. However, since our quantitative TaqMan studies were conducted when the samples were thawed for the first time it is unlikely that fluctuation profiles would have been affected.

In conclusion, we observed several patterns of viral kinetics among PWID with incident HCV infection. The rate of spontaneous clearance was relatively high in the one-year perspective. Incident HCV infection was associated with a decline in HCV viremia, in some cases to levels below the detection limit, at seroconversion to anti-HCV. This shows the importance of HCV RNA follow-up testing in order to avoid misclassification of spontaneous resolution. In addition, prospective testing of people with active injection drug use for both viral antibody and RNA, could allow for identification of persons with acute HCV infection and for further interventions to limit transmission.
